# Advancing the Science of Spatial Neglect Rehabilitation: An Improved Statistical Approach with Mixed Linear Modeling

**DOI:** 10.3389/fnhum.2013.00211

**Published:** 2013-05-20

**Authors:** Kelly M. Goedert, Raymond C. Boston, A. M. Barrett

**Affiliations:** ^1^Department of Psychology, Seton Hall UniversitySouth Orange, NJ, USA; ^2^Clinical Studies, New Bolton Center, School of Veterinary Medicine, University of PennsylvaniaPhiladelphia, PA, USA; ^3^Kessler Foundation Research CenterWest Orange, NJ, USA

**Keywords:** spatial neglect, rehabilitation, mixed linear modeling, statistical methods, power simulation, type I error simulation

## Abstract

Valid research on neglect rehabilitation demands a statistical approach commensurate with the characteristics of neglect rehabilitation data: neglect arises from impairment in distinct brain networks leading to large between-subject variability in baseline symptoms and recovery trajectories. Studies enrolling medically ill, disabled patients, may suffer from missing, unbalanced data, and small sample sizes. Finally, assessment of *rehabilitation* requires a description of continuous recovery trajectories. Unfortunately, the statistical method currently employed in most studies of neglect treatment [repeated measures analysis of variance (ANOVA), rANOVA] does not well-address these issues. Here we review an alternative, mixed linear modeling (MLM), that is more appropriate for assessing change over time. MLM better accounts for between-subject heterogeneity in baseline neglect severity and in recovery trajectory. MLM does not require complete or balanced data, nor does it make strict assumptions regarding the data structure. Furthermore, because MLM better models between-subject heterogeneity it often results in increased power to observe treatment effects with smaller samples. After reviewing current practices in the field, and the assumptions of rANOVA, we provide an introduction to MLM. We review its assumptions, uses, advantages, and disadvantages. Using real and simulated data, we illustrate how MLM may improve the ability to detect effects of treatment over ANOVA, particularly with the small samples typical of neglect research. Furthermore, our simulation analyses result in recommendations for the design of future rehabilitation studies. Because between-subject heterogeneity is one important reason why studies of neglect treatments often yield conflicting results, employing statistical procedures that model this heterogeneity more accurately will increase the efficiency of our efforts to find treatments to improve the lives of individuals with neglect.

## Introduction

Spatial neglect, a deficit in perceiving, orienting, or initiating action toward stimuli in contralesional space (Heilman et al., 2003), affects an estimated one half of right hemisphere stroke survivors annually (Paolucci et al., 2001; Buxbaum et al., 2004; American Heart Association, 2011; Nijboer et al., 2013). Individuals with spatial neglect experience greater disability than do other stroke survivors (Buxbaum et al., 2004; Jehkonen et al., 2006): they have longer hospitalizations (Kalra et al., 1997), poorer rehabilitation outcomes (Gillen et al., 2005), and greater incidence of chronic functional disability (Paolucci et al., 2001). Thus, there is an urgent need to identify therapies that successfully induce recovery of neglect-related cognitive and motor impairment. Our ability to identify these therapies, however, is constrained by the methods we use to assess them.

We argue that the statistical approach typically employed in studies of neglect rehabilitation – repeated measures ANOVA (rANOVA) – is inappropriate given the characteristics of the neglect syndrome and the nature of neglect rehabilitation research. In this research, typically two or more patient groups are assessed prior to the administration of an experimental or control treatment and assessed again one or more times after the treatment. The critical question is whether the amount of change across the assessments is different for the different treatment^1^ groups. Thus, in assessing change across time, rehabilitation research studies are longitudinal studies. We argue that it is time for neglect rehabilitation scientists to join many other psychological scientists in using mixed linear modeling (MLM) for longitudinal data analysis.

Here we first review techniques currently employed in rehabilitation studies of neglect. We then review key characteristics of neglect that critically impact the kind of data rehabilitation researchers encounter, discussing how current techniques fail to adequately address these issues. Finally, we introduce MLM and show how it more appropriately accounts for the inherent variability in the neglect syndrome, allowing for more accurate estimation of treatment-related parameters. Using both real and simulated data, we demonstrate the superior ability of MLM to discriminate recovery trajectories of patient groups relative to rANOVA. Although authors in several fields have deemed MLM superior to rANOVA for most longitudinal and repeated measures data (e.g., Tate and Pituch, 2007; Kwok et al., 2008; Pietrzak et al., 2010; Bernal-Rusiel et al., 2013), neglect rehabilitation researchers have yet to embrace this approach. Our goal in the current paper is to contrast MLM with rANOVA, the analysis technique most frequently used in neglect rehabilitation. Furthermore, in an effort to guide nascent MLM users in the field of neglect rehabilitation, we provide power and Type I error analyses for data structures like those encountered in neglect research. These analyses result in recommendations for the design of future neglect rehabilitation studies.

### Current analysis techniques and their assumptions

The assessment of individuals at multiple points over time leads to nested and correlated data structures: assessments over time are nested within each subject. These measurements taken from the same subject are likely to be more similar to one another than those taken from different subjects (Raudenbush, 2009). Thus, neglect rehabilitation data involves dependent, rather than independent, observations. This dependence among observations renders the use of some statistical procedures such as linear regression or analysis of variance (ANOVA) inappropriate, while other statistical methods such as the dependent samples *t* test, rANOVA, and multivariate ANOVA (MANOVA) may be appropriate under certain circumstances.

We performed a review to assess the current use of statistics in neglect rehabilitation studies: we identified studies for the review via a PubMed literature search using three sets of search terms: “neglect” and “rehabilitation”; “spatial neglect” and “treatment”; and “visual neglect” and “treatment.” We included in our review neglect treatment studies that performed statistical group comparisons of two different neglect treatments, or of a treatment to a control group, or of a group to themselves (e.g., cross-over design), with a minimum of two assessment time-points (minimum pre-post). We included only human rehabilitation studies.

Our review identified 78 studies meeting the above criteria, published between January, 1990, and December, 2012. Table 1 depicts key characteristics of these studies’ design and analyses. As can be seen in the Table, the majority of neglect rehabilitation studies employed rANOVA. The average sample size was 18.11 (SD = 10.58, median = 14.5), but 25% of the studies had total sample sizes of 11 or fewer. Of the 78 studies, 34 studies employed only pre-post measurement (i.e., two measurement waves); 33 employed three measurement waves; 8 employed four waves and three studies employed six waves. Thus, most studies employed rANOVA, had two assessment sessions/measurement waves, and had sample sizes of 15 or less.

**Table 1:** **Status of current data analysis in neglect rehabilitation**.

Statistical technique	Number of studies	Mean sample size (min, max)	Mean measurement waves (min, max)
*t* Test	14	17.1 (4, 39)	2.6 (2, 4)
rANOVA	45	17.9 (4, 40)	2.7 (2, 6)
MANOVA	1	20 (20, 20)	6 (6, 6)
Non-parametric	15	16.5 (10, 30)	2.5 (2, 4)
One-way ANOVA	1	60 (60, 60)	3 (3, 3)
MLM	1	21 (21, 21)	6 (6, 6)
None	1	15 (15, 15)	3 (3, 3)
Total	78	18.1 (4, 60)	2.8 (2, 6)

*rANOVA, repeated measures ANOVA*.

**Figure 1:**
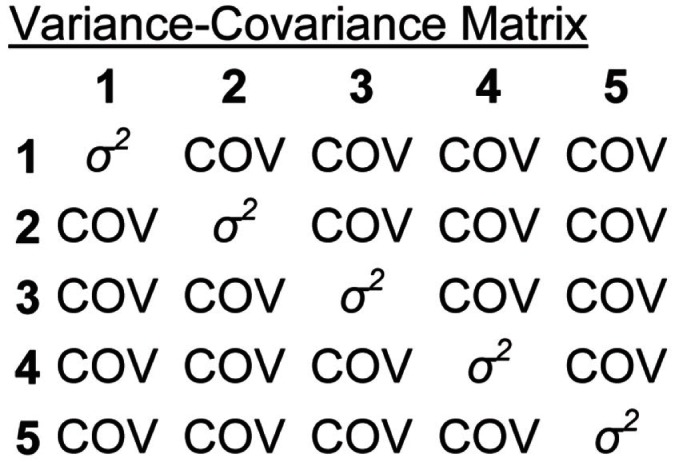
**Variance-covariance matrix depicting homogeneity of variance and compound symmetry assumptions of a repeated measures ANOVA with six repeated assessments**.

Repeated measures ANOVA, the most frequently employed statistical technique, makes three primary assumptions: (1) normality; (2) homogeneity of variance; and (3) either compound symmetry or sphericity (Twisk, 2003). Normality is the assumption that residual variance is normally distributed. Homogeneity of variance is the assumption that variances at all assessment points (and in all groups) are equal. Compound symmetry is the assumption that covariances between all measurement points are equal. Figure 1 represents these latter two assumptions in a variance-covariance matrix for a study with six repeated assessments. The variances at each assessment point are equal (main diagonal) and the covariances between all assessment points are equal (tip: read the Figure like a correlation table, with covariances as squared correlations). A less stringent way of approximating the compound symmetry requirement is the sphericity assumption, which is the assumption that all possible pairs of difference scores between the repeated measures have the same variance (see Rabe-Hesketh and Skrondal, 2012, p. 264, for a more detailed description of compound symmetry vs. sphericity). Data meeting the compound symmetry assumption meets sphericity, but not vice versa. In addition to these assumptions, ANOVA requires complete data, as well as relatively equal samples sizes to ensure homogeneity of variance (Fitzmaurice and Molenberghs, 2009).

Among the other statistical techniques employed in neglect rehabilitation research, MANOVA does not require sphericity or compound symmetry, but it does require normality. Dependent samples *t* tests also require both normality and homogeneity of variance. While non-parametric tests do not entail strict assumptions about the data structure, these are less powerful to detect effects, particularly with violations of homogeneity of variance, and are more limited in their use (e.g., inability to directly test interactions; Siegel and Castellan, 1988; Zimmerman, 1998). Furthermore, similar to ANOVA, the MANOVA, *t* test, and non-parametric test all require complete data, with MANOVA and *t* tests also requiring relatively equal cell sizes (Twisk, 2003; Fitzmaurice and Molenberghs, 2009).

### The neglect syndrome and neglect rehabilitation data

Here we review characteristics of the neglect syndrome that affect the structure of neglect rehabilitation data. Some of these characteristics create particular issues for the neglect rehabilitation researcher, while other characteristics create issues that are common amongst longitudinal patient-based research studies. As we discuss in detail below, rANOVA falls short in handling each of these issues.

#### Between-subject heterogeneity

Working in the area of neglect rehabilitation presents a special challenge: neglect is not a homogeneous disorder. Rather, spatial neglect is a syndrome resulting from disruption in potentially distinct brain networks, leading to diverse impairments, such as object-centered neglect, perceptual-attentional “where” spatial dysfunction, and motor-intentional “aiming” spatial dysfunction, any one of which may or may not be present in a given patient (Na et al., 1998; Barrett and Burkholder, 2006; Hillis, 2006; Verdon et al., 2010; Corbetta and Shulman, 2011). As a result, there is variability across patients both in the type and severity of symptoms prior to treatment, as well as in how those symptoms change over time either with or without treatment (e.g., Hamilton et al., 2008; Manly et al., 2009; Rengachary et al., 2011; Goedert et al., 2012; Nijboer et al., 2013). Thus, neglect rehabilitation demands a statistical approach that accounts for potentially large between-subject heterogeneity among patients both at baseline and in their recovery trajectories.

In rANOVA, variability due to between-subject differences is modeled with the “subjects” term. As a main effect of subjects, it portrays the total variability in the data due to subjects, averaged over the repeated assessments. Thus, while rANOVA models between-subject variability, it does not distinguish between-subject differences in baseline performance from between-subject differences in recovery trajectories (i.e., slope of the change over the repeated assessments). More accurate modeling of these two separate contributions of subjects to the overall variability in the data has the potential to decrease the amount of error variability, thereby improving power to detect treatment effects. However, the ability to do this eludes the researcher who employs rANOVA.

#### Change over time

**Figure 2:**
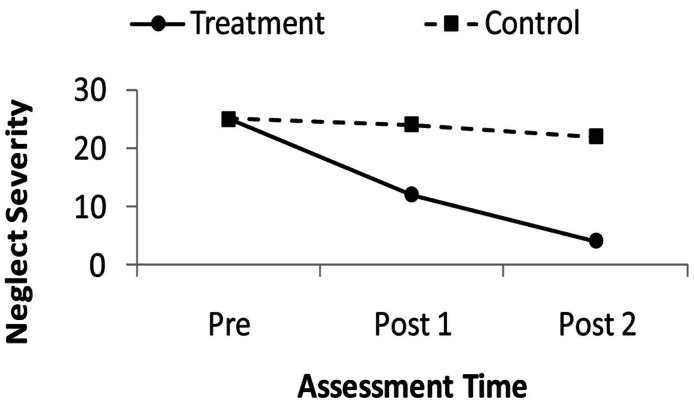
**Depiction of fictional, idealized recovery trajectories in the control, and treatment groups of a neglect rehabilitation study**. Larger values on the *y*-axis indicate more severe neglect.

Although the use of only pre- and post-treatment assessments is very common, evaluating the success of rehabilitation necessitates an interest in change over time – that is, an interest in patients’ recovery *trajectories*. Whether a treatment changes the nature of neglect patients’ recovery trajectories is a question regarding *continuous* development. Figure 2 represents the fictional results of an idealized neglect treatment study in which the severity of neglect in both a control and treatment group have been assessed three times. A key question for neglect rehabilitation is whether the slope of the recovery trajectory in the treatment group differs from that of the control group – that is, whether there is a time by group interaction. Figure 2 represents idealized data as both groups have a similar starting neglect severity and the control group changes little after the treatment (i.e., a very shallow slope on the recovery trajectory), while the treatment group has a steep slope on its recovery over the repeated assessments.

Repeated measures ANOVA does not provide a description of continuous change over time in this situation. In the ANOVA, time is a discrete factor variable, rather than a continuous variable. Thus, were we to analyze the fictional data in Figure 2 and find a significant group by time interaction, we would know that somewhere among the six means (three assessments for each of the two groups) there were significant differences not accounted for by either the main effect of session or the main effect of group. *Post hoc* tests would be needed to determine where those significant differences were (Twisk, 2003; Keppel and Wickens, 2004). Thus, ANOVA does not provide a descriptive value of the magnitude of the change over time, such as the slope value that is produced in linear regression.

#### Violations of compound symmetry and sphericity

Although distinct in a number of respects, neglect rehabilitation research also faces problems common amongst studies of change over time (for a review, see Gibbons et al., 2010). Repeated measures taken from the same subjects are likely to be correlated (Twisk, 2003; Raudenbush, 2009). Furthermore, they are likely to have an auto-regressive covariance structure, such that data-points closer together in time tend to be more correlated with one another than data-points farther away in time. That is, the strength of auto-correlation in the data decreases as time between the assessments increases (e.g., Littell et al., 2000; Fitzmaurice and Molenberghs, 2009). For example, referring to Figure 1, an auto-regressive structure would be apparent if the correlation (or covariance) between measurements taken at time 1 and 2 were higher than that observed between time 1 and 3.

Given that repeated measures taken from the same individuals often have an auto-regressive covariance structure, the assumptions of compound symmetry and sphericity required for rANOVA may be violated in neglect rehabilitation data. Although one can test for violations of sphericity, these tests are sensitive to sample size and are likely to be significant with small violations of sphericity in large samples and, conversely, fail to reach significance with large violations of sphericity in small samples (Twisk, 2003). When the assumption of sphericity is violated, some researchers have turned to employing corrections on the degrees of freedom from the rANOVA (e.g., Greenhouse and Geisser, 1959), or to non-parametric methods. Both options, however, suffer from reduced power to detect treatment effects. Other researchers choose to employ repeated measures MANOVA, which does not entail the compound symmetry or sphericity assumption, but it does require complete data with relatively equal cell sizes (Twisk, 2003; Fitzmaurice and Molenberghs, 2009).

#### Correlations between baseline performance and recovery trajectories

In addition to the likelihood of having an auto-regressive covariance structure, there may be a distinct relationship between neglect patients’ baseline severity and the slope of their recovery trajectories. Although one might expect that the better-off a patient is at baseline, the less room that patient would have for improvement (e.g., Wang et al., 2009), recent studies of spatial neglect demonstrated the opposite: patients better-off at baseline improved more with a prism adaptation treatment than did more severe patients (e.g., Mizuno et al., 2011; Chen et al., 2012; Goedert et al., 2012). Thus, there is an expectation that subjects’ starting point and the slope of their recovery trajectory will be correlated. This correlation is theoretically interesting as it can reveal information about the nature of the neglect treatment (e.g., may only work for less severely impaired patients). Furthermore, this correlation represents systematic variability in the data that can potentially be modeled in an analysis, thereby potentially reducing error variability increasing power. However, it is not possible to model this correlation when using rANOVA.

#### Small sample sizes, missing data, and unequal cell sizes

Similar to other patient-based longitudinal work, neglect rehabilitation researchers face missing data, unequal cell sizes, and small samples. These issues, however, may be particularly exacerbated when studying neglect: stroke survivors with spatial neglect usually have multiple medical conditions and are, as a group, more disabled than other stroke survivors (Buxbaum et al., 2004; Jehkonen et al., 2006; Paolucci et al., 2010). This makes data collection at rigidly fixed intervals very challenging. When subjects miss an assessment due to circumstances outside the researcher’s control (Fitzmaurice et al., 2011), this leads to missing data. Furthermore, neglect is also associated with higher caregiver burden and reduced self-awareness (Buxbaum et al., 2004), which may lead to increased attrition, resulting in unbalanced sample sizes among treatment groups or overall small sample sizes.

### An alternative to ANOVA: mixed linear modeling

Given that as rehabilitation researchers we are interested in change over time, it would be beneficial to adopt a statistical tool developed for the purpose of analyzing change over time. One such tool, MLM or multilevel modeling (also referred to as hierarchical linear modeling, mixed-effects modeling, and random effects analysis), has emerged as a clear alternative to ANOVA for analysis of longitudinal and repeated measures data (see West et al., 2007, for a review). The MLM approach is a regression-based approach that differs from rANOVA in two key respects critical to neglect rehabilitation and other longitudinal research studies: (1) While ANOVA accounts for the correlated structure of repeated measures by modeling a main effect of subjects (i.e., the effect of subjects averaged over the repeated assessments), in MLM one can model subject-level differences in both intercepts (i.e., starting neglect severity) and in slopes (i.e., neglect recovery over time), as well as the correlation between subjects’ intercepts and slopes. (2) With ANOVA, one asks whether any of the repeated measurement points differs from any of the others, but with MLM, one obtains a slope of the recovery trajectory that describes how a patient’s symptoms change over time.

To introduce MLM, let’s take as a starting point the equation for simple linear regression and assume we want to predict neglect severity (*Y_i_*) with assessment time-point as the sole predictor:

(1) $$Y_{i}=\;b_{\text{0}}+b_{\text{1}}\left(\text{assessment}\right)+\varepsilon_{i}$$

Here, *Y_i_* is the predicted *Y* value at time-point *i*, *b*_0_ is the group-level intercept and *b*_1_ is the group-level slope on assessment (it describes the average recovery trajectory across all subjects), and ε*_i_* is the residual error variability at time-point *i*. Because this is a regression analysis, assessment time in Eq. 1 is treated as a continuous predictor. Standard regression, however, assumes independence of observations. It is therefore not appropriate for repeated measures data, such as the repeated assessment of neglect over time. In contrast, MLM is appropriate for repeated measures data.

Although it is a regression-based model, MLM accounts for the dependencies in repeated measures data by separately modeling variability due to subjects, with the option to do so for both between-subject differences in intercepts and between-subject differences in slopes. These subject effects are termed *random effects*. In the case of repeated assessments within subjects, this separate modeling of subject effects occurs via the creation of two levels of regression equations. At the highest level is a regression equation that describes the group-level intercept and slope, as depicted in Eq. 1. This group-averaged intercept and slope are the *fixed effects* in the MLM analysis. At the lowest level in the MLM analysis are subject-specific regression equations representing the random effect of subjects^2^. At this level, the predicted *Y* differs for each subject *j* such that:

(2) $$Y_{ij}=\text{ (}b_{\text{0}}+b_{0j})+\left(b_{\text{1}}+b_{1j}\right)\left(\text{assessment}\right)+e_{\mathrm{ij}}$$

Here, *Y_ij_* is the predicted *Y* value at time-point *i* for subject *j*, *b*_0_ is the group-level intercept, *b*_0*j*_ is the *difference* between subject *j*’s intercept and the group-average intercept, *b_1_* is the group-level slope, and *b*_1*j*_ is the *difference* between-subject *j*’s slope and the group-average slope. Thus, taking Eqs 1 and 2 together, MLM models variability in the intercept and slope as averaged over the group (fixed effects), and it models variability due to individual differences around the group intercept (random intercept), as well as variability due to individual differences around the group slope (random slope). The MLM model can also be constructed so as to estimate the observed correlation between subjects’ intercepts and slopes. Although computationally the MLM analysis builds an individual regression equation for each subject, the typical output from statistical packages running MLM analyses provides summary terms for the random intercept and slope, reporting the amount of variance in the data due to these effects. Additional analysis commands can be used to extract the subject-level regressions.

What is the purpose of modeling these random effects? A researcher may desire to model these subject-level random effects because of an interest in the individual variability in its own right. For example, as stated earlier, in the case of prism adaptation treatment for neglect, there appears to be a negative correlation between patients’ starting severity (i.e., their intercept) and their response to treatment (i.e., the slope of their recovery trajectory over time; Chen et al., 2012; Goedert et al., 2012). Conversely – or additionally – a researcher may be interested in modeling subject-level random effects as a means of potentially reducing error variability in the statistical analysis, with the possibility of improving power to detect group-level treatment effects (Gueorguieva and Krystal, 2004; Brown and Prescott, 2006; Fitzmaurice et al., 2011). For the neglect rehabilitation researcher, finding a treatment that works at the group-level is the likely goal of this analysis. Thus, the main focus of interpretation in neglect rehabilitation would likely be on the fixed effects (i.e., group-level effects).

#### Assumptions and decisions when using MLM

Mixed linear modeling is not without its own assumptions. Standard MLM assumes normality in residuals of the fixed and random effects. It assumes homogeneity of variance at all levels of the model, and, like simple linear regression, it assumes a linear relation between the predictor and outcome (Singer and Willett, 2003). While a number of these assumptions are similar to those of rANOVA and MANOVA, with MLM it is possible to modify the standard analysis to accommodate violations of these assumptions.

Indeed, unlike rANOVA, when performing an MLM, the researcher *must* make a number of decisions for how to structure the analysis. One such decision is with regards to the structure of the residual covariance matrix: the residual variability represented by the terms ε and *e* in Eqs 1 and 2 refer to residual covariance structures (i.e., structures similar to that depicted in Figure 1). When performing an MLM, the researcher must decide *whether* to impose assumptions on the covariance structure and what assumptions to impose. For example, one could assume an auto-regressive covariance structure (as described in Violations of Compound Symmetry and Sphericity). A number of different covariance structure choices are available in statistical packages. Alternatively, the researcher could decide to make no assumptions about the residual covariance structure, estimating the covariance directly from the data, thereby rendering homogeneity of variance and other assumptions about the variance-covariance structure unnecessary (e.g., Littell et al., 2000).

Although basic MLM makes assumptions of linearity and normality, like other regression models, the researcher has the option to build non-linear relations into the MLM (e.g., polynomial trends, linear splines; Littell et al., 2000; Singer and Willett, 2003; Twisk, 2003; Davidian, 2009). With MLM (as with regression) non-normality may be accommodated via bootstrapping the standard errors of the intercept and slope parameters (Guan, 2003). Thus, MLM models allow for a better match between the model assumptions and the actual data typically observed in neglect rehabilitation and other longitudinal studies.

However, in MLM one must decide how to evaluate significance of the parameter estimates – i.e., how to assess significance of the fixed intercept and slope. Although assessing the significance of terms in the rANOVA is typically straightforward, the researcher deciding to use a degrees of freedom correction for violations of sphericity (e.g., Greenhouse–Geisser or Huynh–Feldt) is making a decision about how to assess significance. In MLM the primary issue is with regards to estimating degrees of freedom, and different statistical packages provide different options and defaults. For example, in STATA and Mplus, Wald’s *z* is the method for assessing significance, which assumes infinite degrees of freedom. Thus, it is only appropriate for large samples. SPSS and SAS assess significance of fixed effects using an *F* distribution. They offer different options for computing degrees of freedom for the *F* test, all of which take into account the size of the sample and number of repeated observations in the analysis. Finally, one must decide whether to use a maximum likelihood estimation procedure or restricted maximum likelihood for the MLM analysis. (A complete discussion of these latter two issues is beyond the scope of the current paper; for a thorough discussion of both, the reader is directed to West et al., 2007, pp. 25–29, 36–38, and 110–113).

#### Advantages of MLM and a longitudinal modeling approach

##### Greater power with smaller samples

Because of the immediate, urgent need to move new therapies forward into widely available clinical practice guidelines, neglect rehabilitation research requires an approach that can make use of smaller sample sizes than those required for typical parametric analysis. Because MLM considers the correlated and nested data structure inherent in measuring the same subjects repeatedly, it results in a much more accurate estimation of variance to calculate between-subject treatment effects (Fitzmaurice et al., 2011). Modeling random intercepts and slopes can result in reduced standard errors for the estimates of the fixed effects (Littell et al., 2000; Gueorguieva and Krystal, 2004; Brown and Prescott, 2006)^3^. Thus, MLM can result in greater power to detect group-level differences, as well as in more narrow confidence intervals around the group-level parameter estimates.

##### Description of the recovery trajectory

A second benefit of the MLM analysis is that it allows us to describe recovery trajectories of treatment and control groups. Again, referring to the fictional data depicted in Figure 2, using MLM we could assess the group by time interaction and if we determine it is significant, produce separate group-level slopes for our treatment and control groups. Thus, MLM, like linear regression, yields a metric that describes the magnitude of change over time in our two groups.

##### Flexible model-building and analysis

An additional benefit of the MLM analysis is that one can readily examine the effects of controlling for additional nuisance variables that may happen to be continuous rather than categorical (e.g., differences in baseline status) or that may be time-varying as opposed to constant across the assessment time-points (Rabe-Hesketh and Skrondal, 2012). Furthermore, these analyses can be conducted while controlling for potential interactions between the continuous predictors and the recovery trajectories (e.g., Cnaan et al., 1997). For example, one could ask whether there were differences in the group-level recovery trajectories while controlling for any improvements across the sessions that may be attributable to baseline status (i.e., while controlling for a baseline status by assessment-session interaction). Although rANOVA and MANOVA can accommodate continuous covariates, one cannot use ANOVA and MANOVA to examine complicated interactions among continuous and factor predictors or among two or more continuous predictors (Twisk, 2003).

##### Good tolerance for missing data and unequal cell sizes

Mixed linear modeling is tolerant of both unequal cell sizes (i.e., unbalanced data) and data that are missing at random (Laird and Ware, 1982; Quene and van den Bergh, 2004; Kwok et al., 2008; Skrondal and Rabe-Hesketh, 2008; Molenberghs and Fitzmaurice, 2009; Gibbons et al., 2010). This relative robustness in the face of missing and unbalanced data results from characteristics of the MLM analysis: (1) treating time as a continuous rather than a factor variable (Kwok et al., 2008) and (2) using maximum likelihood estimation, which entails finding the set of parameter estimates that maximizes the likelihood of the data, rather than least squares estimation, as employed in ANOVA.

In sum, MLM meets the demands of neglect rehabilitation research: it accounts for the between-subject heterogeneity in baseline and recovery that is expected given the distinct brain networks potentially contributing to the neglect syndrome. It affords greater power and it is tolerant of missing and unbalanced data.

## Demonstration and Simulation Analyses Using MLM vs. Repeated Measures ANOVA

In this section we compare the performance of MLM and rANOVA. We start with a re-analysis of a set of our own published data (Chen et al., 2012), comparing the results using an MLM analysis to those using rANOVA. Next, we use simulation methods to compare the power and Type I error rates of MLM and ANOVA under a variety of conditions facing researchers in the field of neglect rehabilitation (i.e., varying sample sizes, varying effect sizes, different number of assessment sessions). Although other simulation studies have compared the power and Type I error rates of MLM and rANOVA (e.g., Gueorguieva and Krystal, 2004; Maas and Hox, 2005), these studies have simulated minimum sample sizes of 20, 30, or even 50, all of which are larger than the average study of neglect patients, whose median sample size is 14.5 (Table 1). Furthermore, previous simulation studies have assumed a zero correlation between subjects’ intercepts and slopes – a situation uncharacteristic of neglect rehabilitation data (Mizuno et al., 2011; Chen et al., 2012; Goedert et al., 2012). Thus, to confirm that MLM is indeed more powerful than rANOVA for neglect rehabilitation data, without a concomitant increase in Type I error rates, we performed a set of simulations generating power and Type I error rates for conditions likely to be encountered by the neglect rehabilitation researcher.

For both the real and simulated data, we assume a study in which we have two groups, each measured over time. Thus the full-factorial analysis includes the main effects of group and assessment time-point as well as their interaction. Here, we focus on the power of the analyses to detect the treatment group by assessment-session interaction, because the key focus of neglect rehabilitation studies would be to detect group *differences* in change over time. All analyses and data simulation were performed using STATA/IC 12.1.

### Re-analysis of published Chen et al. (2012) data

We first turn to a set of data described in a recent study published by members of our research group (Chen et al., 2012). Capitalizing on work demonstrating an association between motor-intentional neglect symptoms and the frontal cortex (e.g., Ghacibeh et al., 2007), as well as work demonstrating that prism adaptation improves motor-intentional, but not perceptual-attentional, neglect (Striemer and Danckert, 2010; Fortis et al., 2011) we expected that patients with frontal lesions might experience more improvement with prism adaptation treatment than those without frontal lesions. Twenty-one right brain-damaged subjects with left spatial neglect underwent 2 weeks of prism adaptation treatment (once daily for 5 days per week). We assessed subjects’ neglect with the Catherine Bergego Scale (CBS) just prior to the start of prism adaptation treatment and weekly thereafter for 5 weeks. We used subjects’ clinical CT or MRI scans to map their lesions and categorized subjects as having the presence (*n* = 13) or absence (*n* = 8) of a frontal lesion. Although the original study reported a more complicated MLM analysis, here we focus on a simple analysis including the predictors of frontal lesion (present, absent), assessment session (one through six), and the frontal lesion by assessment-session interaction.

#### Analyses and results

For the MLM we modeled the frontal lesion by assessment-session factorial as fixed effects (with assessment session as a continuous variable) and we modeled subjects’ intercepts and slopes as random effects. Because we were primarily interested in the fixed effects, we used maximum likelihood estimation, which provides more accurate estimates of the fixed effects than does restricted maximum likelihood, which may better-model random effects (West et al., 2007). We used an unstructured covariance matrix for the random effects, which meant our analysis could estimate a correlation between the random intercepts and slopes. And, we used a residual covariance matrix that assumed homogeneity of variance across the assessment sessions. Although STATA reports Wald’s *z* for evaluating the significance of the fixed effects, we report the results of *F* tests, calculated using between-within degrees of freedom (West et al., 2007). As mentioned previously, use of *z* assumes a large sample, and may overestimate the significance of fixed effects. Therefore, we assessed their significance using the same *df* and *F* distribution that would be used in the comparable mixed between-within ANOVA. Results of this MLM analysis are depicted in Table 2.

**Table 2:** **Results of MLM analysis of Chen et al. (2012) data**.

	*b*	SE	95% CI	*F* test
**Fixed effects**				
Session	−0.63	0.42	−1.46, 0.19	*F*(1, 86) = 2.25, *p* = 0.137
Frontal lesion	−2.39	4.06	−10.34, 5.56	*F*(1, 19) = 0.35, *p* = 0.562
Session × lesion	−1.17	0.53	−2.21, −0.13	*F*(1, 86) = 4.88, *p* = 0.030
**Random effects**				
SD (slope on session)	0.927	0.24	0.56, 1.53	NA
SD (intercept)	8.423	1.51	5.93, 11.96	NA
Corr (int, slope)	−0.933	0.05	−0.99, −0.71	NA
**Residual SD**	2.56	0.22	2.16, 3.03	NA

For the rANOVA we modeled the full factorial of frontal lesion (presence, absence) and assessment session (one through six), with assessment session as a discrete, factor variable. The test of sphericity was significant, *p* < 0.001, indicating neither the compound symmetry nor sphericity assumption was met in this set of data. Table 3 depicts the results of the rANOVA and, given the violation of sphericity, Greenhouse–Geisser corrected *p*-values.

**Table 3:** **Results of repeated measures ANOVA of Chen et al. (2012) data**.

Source	Partial SS	*df*	*F* test	G–G corrected *p*
Frontal lesion	1142.59	1	*F*(1, 19) = 8.44, *p* = 0.009	
Subjects	2572.08	19		
Session	451.20	5	*F*(5, 78) = 9.68, *p* < 0.001	*p* < 0.001
Session × lesion	89.74	5	*F*(5, 78) = 1.92, *p* = 0.099	*p* = 0.185
Residual	727.77	78		

Comparing across analyses, we see that the MLM detected a significant lesion by session interaction, while the ANOVA did not. Inspection of the group-level slopes from the MLM revealed that the group without frontal lesions had a slope on their recovery trajectory that did not differ significantly from zero, *b* = −0.63, SE = 0.13, 95% CI [−1.46, 0.19], while the group with frontal lesions showed significant improvement across the assessment sessions, *b* = −1.80, SE = 0.32, 95% CI [−2.42, −1.18]. Conversely, the ANOVA indicated significant main effects of session and presence of frontal lesion, while the MLM did not. Note that for the ANOVA, the effect of session indicates that at least one of the six assessment sessions significantly differs from another. In contrast, for the MLM, the non-significant effect of session signifies that, controlling for the group by session interaction, the group-average linear slope on session was not significantly different than zero.

The significant main effect of presence vs. absence of frontal lesions for the ANOVA, but not the MLM, suggests that this effect might be an artifact of the random effects structure of the data that is accounted for by the MLM but not by the ANOVA. In particular, note that with the MLM we have estimated the variability due to individual differences in subjects’ slopes from the group slope (SD for slope on session) as well as the variability due to individual differences in subjects’ intercepts from the group intercept (SD on intercept). Finally, the MLM estimates the correlation between the subjects’ intercepts and slopes. Because lower scores on the CBS indicate less severe neglect, this negative correlation of −0.93 indicates that subjects with less severe neglect at baseline demonstrated greater improvement across the assessment sessions.

We see from this re-analysis of the Chen et al. (2012) data that MLM was better-able to detect a difference between the recovery trajectories of the groups with and without frontal lesions. Additionally, the MLM analysis described the continuous change in the data with the slope values: across the six assessments, the group with frontal lesions improved an average of 1.80 points on the CBS per week, while the group without frontal lesions improved an average of 0.63 points per week.

### Simulation study

In order to compare the power and Type I error rates of MLM and rANOVA under varying circumstances likely to be encountered by neglect rehabilitation researchers, we performed a Monte Carlo simulation study: we repeatedly generated data sets and performed MLM and rANOVA analyses on each of the generated datasets. In total, we performed 24 simulations in which we generated data sets that varied in sample size (*N* = 6, 20, or 30), the number of assessment sessions/measurement waves (3 or 6), and effect size. Here we chose to present the simulation of 3 and 6 measurement waves because a minimum of three assessment points is considered critical for the assessment of change over time (see Discussion). Furthermore, the focus of our simulation was on the ability to detect a group by assessment-session interaction, because this would indicate a difference in response to treatment in the two groups. Because we were interested in the interaction, we measured effect size as the standardized difference between the slopes of the two groups (*d* = 0.20, 0.50, 0.80, or 1.00; assuming a SD on the group-level slopes of 2.00). Finally, we assessed Type I error rates with simulations in which the standardized difference between the group slopes was zero. Table 4 summarizes the group-level fixed effect slopes used in the data-generation process at each effect size. The simulation yielded estimates of power and Type I error. These estimates indicate the proportion of samples we would expect to achieve significance at a significance level of 0.05. We provide these estimates for the repeated measures ANOVA (rANOVA), the Greenhouse–Geisser corrected rANOVA (GG-ANOVA), the Wald’s *z* test of the MLM fixed effects (MLM-*z*), and the *F* test of the MLM fixed effects (MLM-*F*). A complete description of the simulation method appears in the Appendix.

**Table 4:** **Summary of group-level fixed slopes for putative “control” and “treatment” groups at each simulated effect size**.

	Group	
Effect size (*d*)	Control	Treatment
0.20	0.00	−0.40
0.50	0.00	−1.00
0.80	0.00	−1.60
1.00	0.00	−2.00
0.00	0.00	−0.00

#### Simulation results and discussion

##### Three assessments/measurement waves

**Figure 3:**
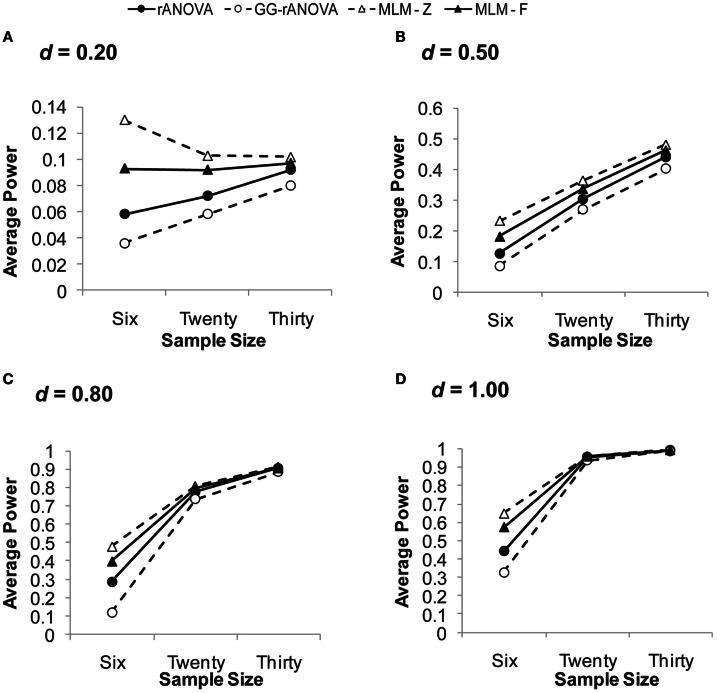
**Average power on the session by group interaction with three measurement waves**. **(A)** for *d* = 0.20; **(B)** for *d* = 0.50; **(C)** for *d* = 0.80; **(D)** for *d* = 1.00. *d*, standardized difference between group slopes. rANOVA = repeated measures ANOVA, GG-rANOVA is Greenhouse–Geisser corrected repeated measures ANOVA, MLM-*Z* is Wald’s *Z* from the MLM, MLM-*F* is the between-within *df* for *F* from the MLM.

Figure 3 depicts the power to detect the group by session interaction with three measurement waves. Looking across effect sizes, it is clear that the MLM (for both *z* and *F*) has superior power to the ANOVA, particularly at smaller sample and smaller effect sizes. The rANOVA with the Greenhouse–Geisser correction has the poorest power, except at large effect and large sample sizes. For example, for an effect size of *d* = 0.20, the GG-rANOVA would reach significance less than 4% of the time for samples of size 6 and only 6% of the time for samples of size 20. Conversely, the MLM using Wald’s *z* (MLM-*z*) has greater power than the other estimates, except where there is convergence among all the measures for the effect size of *d* = 1.00 with at least 20 subjects.

**Figure 4:**
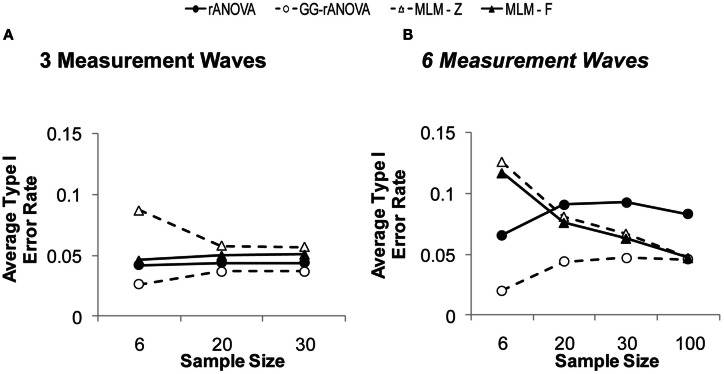
**Type I error rates on the session by group interaction with (A) three and (B) six measurement waves**.

But a complete picture of these measures’ performance requires an inspection of their concomitant Type I error rates, which are depicted in Figure 4A. Given the choice of 0.05 as the significance level, the extent to which any of the estimates shows a Type I error rate greater than or less than 0.05 suggests bias in the statistical test. As can be seen in Figure 4A, the MLM using Wald’s *z* shows unacceptable levels of Type I error at a sample size of six. Although this rate of Type I error reduces at larger sample sizes, it still hovers just below 0.06, likely due to the large sample assumption of the *z* distribution. Thus, our results confirm the inappropriateness of Wald’s *z* for smaller sample sizes.

The MLM-*F* has a Type I error rate that is just below 0.05 for the smallest sample size and right at 0.05 for samples of size 20 and 30. Thus, for three measurement waves, the MLM-*F* does not show bias. The rANOVA, however, remains below 0.05 across sample sizes, and thus, shows a slight conservative bias, which would lead to Type II errors (i.e., failure to detect a real effect). The GG-rANOVA is even more conservatively biased than is the rANOVA: it too would lead to Type II errors.

In sum, with three measurement waves, the MLM using the *F* distribution with between-within degrees of freedom (West et al., 2007) shows good power, while also showing no bias in Type I error rates. This result is consistent with other simulation work showing that MLM performs well in estimating fixed effects with few repeated measurements (Bell et al., 2010). We extend this previous work by showing the superiority of the MLM even for very small sample sizes (*N* = 6), as long as one uses the *F* distribution for assessing significance.

##### Six assessments/measurement waves

**Figure 5:**
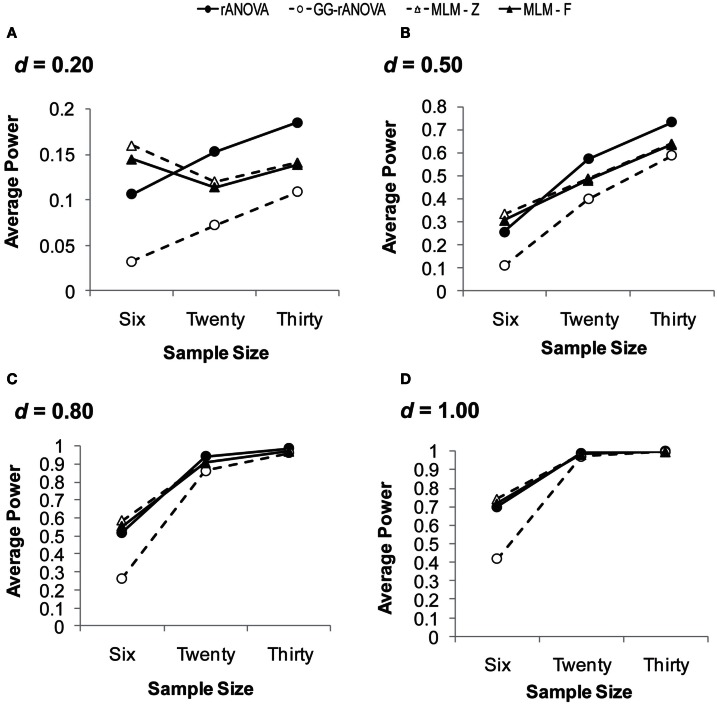
**Average power on the session by group interaction with six measurement waves**. **(A)** for *d* = 0.20; **(B)** for *d* = 0.50; **(C)** for *d* = 0.80; **(D)** for *d* = 1.00. *d* = standardized difference between group slopes, rANOVA = repeated measures ANOVA, GG-rANOVA is Greenhouse–Geisser corrected repeated measures ANOVA, MLM-*Z*, Wald’s *Z* from the MLM, MLM-*F* is the between-within *df* for *F* from the MLM.

Figure 5 depicts the power to detect the group by session interaction with six measurement waves. Looking in particular at the small effect sizes, we see a pattern that is very different from that observed with three measurement waves. With sample sizes of 20 and 30, the rANOVA demonstrates superior power to the other three statistics. Consistent with the pattern observed with three measurement waves, the Greenhouse–Geisser corrected ANOVA shows poor power except with larger samples and large effect sizes. Similar to what was observed with three measurement waves, the estimates of all the analyses converge with samples of at least 20 at the largest effect size (*d* = 1.00).

Again, for a complete picture of the analyses’ performance we must inspect their Type I error rates, which are depicted in Figure 4B. Figure 4B depicts not only the simulations described above, but an additional simulation of the Type I error rates in a sample size of 100. In the Figure, we can see that both MLM-*z* and MLM-*F* are biased at smaller sample sizes, with Type I error rates well above 0.05 at a sample size of six. However, this bias reduces as the sample size increases, with the Type I error rate converging on 0.05 at larger sample sizes. Conversely, for the rANOVA, Type I error rates remain unacceptably large even at the largest sample size of 100. The Greenhouse–Geisser corrected rANOVA shows conservative bias at the smaller sample sizes, but like the MLM estimates, its Type I error rates converge on 0.05 at the larger sample sizes.

##### Simulation summary

The Monte Carlo simulation allows us to compare the performance of MLM and rANOVA with different sample and effect sizes. In sum, the simulation study demonstrates that overall, MLM using Wald’s *z* creates too much Type I error, while the rANOVA using the Greenhouse–Geisser correction is too conservative, sacrificing too much power. The simulation study further demonstrates that with three measurement waves, the MLM-*F* has superior power and is unbiased, while the rANOVA has poorer power and is conservatively biased (i.e., Type I error rates less than 0.05). Thus, with three repeated assessments, the MLM-*F* is better-able to detect treatment effects without an increase in Type I error rates. With six measurement waves, the ANOVA has more power at smaller sample sizes, but also has an unacceptably high rate of Type I error at *all* samples sizes (i.e., even at *N* = 100 the Type I error rate of rANOVA is at 0.08). Conversely, the Type I error rates of the MLM converge on 0.05 at larger sample sizes. Thus, it appears that with six repeated assessments, the MLM more accurately estimates the fixed effects with increases in sample size; whereas the accuracy of the rANOVA does not systematically increase with increases in sample size.

## General Discussion

We argued that neglect rehabilitation demands a statistical approach commensurate with characteristics of the neglect syndrome. In the re-analysis of our previously published data we demonstrated how MLM provides a description of the recovery trajectory and how it was better-able to detect the difference in the recovery trajectories of the groups with and without frontal lesions. Furthermore, the difference between the MLM and rANOVA analyses suggest that the ANOVA may have inappropriately modeled variability arising from individual differences in recovery trajectories.

The results of our simulation suggest that MLM does indeed have superior power at smaller sample sizes, and that it can be confidently used with the small samples often employed in neglect rehabilitation research if analyzing a small number of measurement waves. With many measurement waves (in this case six), larger sample sizes may be needed for MLM to accurately estimate the fixed effects. This result is consistent with the observations of others (Snijders, 2005), that accuracy of estimation of the fixed effects is primarily driven by the size of the sample at that level of estimation. Furthermore, our results likely overestimate the ability of ANOVA relative to MLM, given that we simulated complete and balanced data structures. Relative to ANOVA, MLM has the added advantage of continuing to perform well even when data are missing at random (Gueorguieva and Krystal, 2004; Quene and van den Bergh, 2004), a situation likely encountered by researchers of neglect rehabilitation.

### More than analysis tool: Longitudinal modeling as a research approach

Perhaps more than just the benefits afforded by a potentially more powerful and more appropriate statistical tool, a look at using MLM could help neglect rehabilitation researchers better-conceptualize their research problem. As researchers interested in the rehabilitation of patients with neglect, we must be interested in how individuals change over time and how we can alter those recovery trajectories with rehabilitative treatment. Thus, we need a statistical tool that allows us to describe those trajectories.

However, the ability to describe recovery trajectories is related not just to the statistical approach, but also to the study design: the majority of the treatment studies that we reviewed measured patients’ performance at only two time-points: once before treatment and once immediately after treatment. However, use of only two assessment points pre- and post-treatment confounds true change and measurement error (Rogosa et al., 1982; Singer and Willett, 2003). Furthermore, a simple pre-post difference does not provide a picture of how patients’ change over time because it tells us nothing of subjects’ individual recovery trajectories (Singer and Willett, 2003). For example, are recovery trajectories linear or quadratic? Are the benefits of a treatment experienced immediately and then level off, or do benefits of the treatment continue as time post-treatment increases? Assessing neglect patients at a minimum of three measurement waves will help answer these important questions about neglect recovery and its relation to rehabilitation.

### Limitations and barriers in the use of MLM

Despite the potential advantages of MLM, it is not without its own limitations. First, although bootstrapping procedures may be used to overcome violations of normality in the distribution of the residuals, the use of bootstrapped standard error estimates may restrict the researcher to less complicated forms of MLM analysis (e.g., modeling only random intercepts, without modeling of random slopes). Second, MLM models do not always resolve. That is, the maximum likelihood estimation process may not converge on a set of parameter estimates or may be unable to estimate standard errors. This is more likely to happen with smaller sample sizes and with more complex models. Thus, under certain circumstances, the researcher may be restricted to using a simpler MLM model (e.g., may have to assume homogeneity of variance rather than modeling an auto-regressive residual covariance structure).

Finally, MLM procedures are statistically more complex than is rANOVA. However, MLM procedures are now integrated into major statistical packages including SPSS, SAS, STATA, and R. It must be recognized that some of the statistical complexity comes with the added benefit of greater flexibility in the analyses, such as the ability to model alternate residual structures so as to avoid violations of assumptions like homogeneity of variance (Cnaan et al., 1997).

### Limitations in the current treatment of MLM

Our discussion of MLM in the current paper is necessarily limited by our desire to present a simple introduction to MLM for the neglect rehabilitation researcher who is likely to be currently using rANOVA. As a result, there are several issues of importance in using MLM to analyze longitudinal data from treatment (and other) studies that were beyond the scope of this paper.

First, it is standard when performing MLM to quantifying the amount of nested dependency in a dataset by calculating the intraclass correlation coefficient (ICC). In the case of repeated assessments nested within subjects, the ICC is the proportion of total variance in the data that is accounted for by between-participant differences (Singer and Willett, 2003). ICCs at or close to zero suggest that the data are actually independent rather than dependent, and that modeling of subjects’ random effects is unnecessary.

Second, the models we presented, both for the fictional data (Figure 2) and for the re-analysis of the Chen et al. (2012) data, were necessarily simplified. In a neglect treatment study, MLM would allow the researcher to control for and assess additional factors affecting neglect recovery, such as baseline severity. Indeed, in the previously published Chen et al. (2012) analysis, we controlled for patients’ spontaneous recovery rates as estimated by the slope of their recovery trajectories prior to initiating a prism treatment.

Third, in this paper, we focused primarily on the performance of MLM on fixed effects estimation – that is, the group-level intercepts and slopes – and in particular, on the group by session interaction. This focus does not do justice to the full potential of the MLM analysis, particularly for modeling individual change over time. As we saw in the re-analysis of our previously published data, MLM provides estimates not just of fixed effects, but also of the variability due to the random effects, and the correlation between the random intercept and slope. Additionally, one can also model cross-level interactions, as well as examine individual-level trajectories (e.g., Cnaan et al., 1997; Rabe-Hesketh and Everitt, 2003). None of the power and Type I error simulations we performed here may be generalized to the subject-level random effects. Power and precision of the random effects estimation is largely driven by the sample size at that level (Snijders, 2005).

Indeed, MLM may be appropriate for many study designs involving dependencies among measures (e.g., longitudinal, repeated measures, and clustered data) with outcomes that are either continuous, binary, or ordinal (Rabe-Hesketh and Skrondal, 2012). A full discussion of its uses, however, is beyond the scope of the current paper. West et al. (2007) provide a good treatment of the use of MLM in different study designs. Furthermore, Liu et al., 2012 provide a comprehensive discussion of how to decide among analyses for use with longitudinal data. They argue that rANOVA might be more appropriate when the researcher wishes to treat time as a factor variable. However, in an MLM time can be treated as a factor variable. Indeed, rANOVA may be thought of as a special case of MLM – one in which the residual variance-covariance matrix assumes both homogeneity of variance and sphericity and in which the only random effect modeled is the random intercept (i.e., the subjects term in the ANOVA is analogous to the random intercept of the MLM).

### Recommendations for neglect researchers starting in MLM

Our simulation results lead us to make several recommendations for neglect rehabilitation researchers:

(1) If using three assessment sessions, MLM offers more power than rANOVA, particularly at the small samples sizes typical of neglect rehabilitation research.
(2) If using six assessment sessions, rANOVA has high Type I error rates even at large sample sizes, while MLM performs well as sample size increases. Thus, if using many repeated assessment sessions, rANOVA should not be used and the use of MLM will require a larger sample size (e.g., 30 or more) for valid statistical inference on the fixed effects.
(3) The default means of assessing significance of the MLM fixed effects parameters in STATA and Mplus (Wald’s *z*) should not be used with the small samples typical of neglect rehabilitation studies. Rather, the *F* distribution should be used for assessing the significance of these effects.

Several resources are particularly useful for researchers getting started in using MLM. Andy Field provides a very accessible first-introduction to performing MLM analyses, with chapters dedicated to MLM in his books on SPSS and R (Field, 2010; Field et al., 2012). West et al. (2007) is an excellent introduction to performing various types of MLM analyses, illustrating the analyses in R, SPSS, SAS, and STATA. Rabe-Hesketh and Skrondal (2012) is an authoritative and thorough examination of MLM for longitudinal data structures in STATA. Finally, both Fitzmaurice et al. (2011) and Singer and Willett (2003), provide comprehensive conceptual treatments of using MLM for longitudinal data analysis.

## Conclusion

Neglect rehabilitation research demands a statistical approach commensurate with the characteristics of the neglect syndrome. Given that neglect arises from disruptions to potentially distinct brain networks and results in disparate patterns of behavioral symptoms, the field requires a statistical technique designed to adequately account for between-subject variability in baseline status and recovery trajectory. Further, the study of neglect rehabilitation requires a technique that allows the researcher to describe patients’ change over time. MLM meets both these demands of neglect rehabilitation data. MLM offers the additional advantage of superior power at small sample sizes, and it does not require complete data.

Given its power and Type I error rate, and given its robustness in the face of missing data, we think MLM the ideal tool for analyzing data from neglect rehabilitation studies. We look forward to the future of neglect rehabilitation research when, hopefully, it will be more common to find 3+ measurement waves and when multilevel modeling to investigate patient change over time is the new standard.

## Conflict of Interest Statement

The authors declare that the research was conducted in the absence of any commercial or financial relationships that could be construed as a potential conflict of interest.

## Footnotes

^1^In the current paper, the terms *treatment* and *rehabilitation* are used interchangeably, given that the goal of most neglect treatment studies is rehabilitation. However, these two terms can have distinct meanings: treatment sometimes refers to interventions designed to address only symptoms of a disorder, while rehabilitation consistently refers to interventions designed to ameliorate the underlying cause of symptoms.

^2^Note, in MLM terminology, the random effects of subjects, as described here, would be considered level-1 effects and the group-level fixed effects would be considered level-2 effects. Whether “subjects” are the level-1 or level-2 effects depends, however, on the study design. In a study of patients nested within hospitals, patients would be the lowest level (i.e., level-1) and hospitals would be the higher level (i.e., level-2; see Chapter 1 of West et al., 2007, for a comparison of study designs appropriate for MLM and a description of what constitutes the levels in those designs). One could also create a 3-level model with multiple measurement points (level-1) nested within patients (level-2), which themselves are nested within hospitals (level-3).

^3^This is not a necessary effect of modeling the random effects (Rabe-Hesketh and Skrondal, 2012), but frequently results when modeling the random effects for data with large between-subject variation relative to within-subject variation (discussed by Brown and Prescott, 2006; Fitzmaurice et al., 2011; Gueorguieva and Krystal, 2004). It is a result we have observed in our own MLM analyses of neglect rehabilitation data, likely due to large between-subject variation. That is, adding random effects to an MLM model that first contained fixed effects typically does not change the fixed effect coefficients, but it reduces the standard error on those coefficients. However, as discussed by Rabe-Hesketh and Skrondal (2012, pp. 167–168), the exact result of introducing random effects into the MLM also depends on whether the fixed effect factor is completely within-cluster (i.e., within-subjects), as time is when assessing change over time, or whether the fixed effect factor is completely between-cluster. For within-cluster fixed effects, adding specification of the random effects can reduce the standard errors of the fixed effect parameter estimates (relative to ordinary least squares regression). The opposite can occur for completely between-cluster fixed effect factors. Furthermore, the magnitude of these changes varies with sample size (Snijders and Bosker, 1993, p. 253). Other factors affect the fixed effects standard errors, such that when the sample size is small, the data are not balanced, and between-subject variability is small relative to within-subject variability, the estimated standard errors may be too small (i.e., a biased estimate of the variance) and corrections may be necessary when significance testing (Brown and Prescott, 2006, pp. 75–76; Kenward and Roger, 1997).

## Appendix

### Data-generation process for simulation

We used the random effects model depicted in Eq. 2 as the underlying data-generating model, creating different equations for the “control” and “treatment” groups. We derived parameters from which to generate the random effects and residual variability by averaging the variability due to subjects’ intercepts, their slopes, and their residuals over the Chen et al. (2012) data and other data from our lab (Goedert et al., 2012).

To generate each data set, we first set the group-level slopes for the control and treatment groups per Table 4. We introduced slightly variability in the group-level intercept, as we had observed in our own data, setting that of the control to 19.07 and that of the treatment group to 21.12. We then generated each subjects’ deviation around the mean intercept and slope (i.e., *b*_0*j*_ and *b*_1*j*_ of Eq. 2) by randomly drawing values from a normal distribution with means of 0 and a standard deviation of 8.5 for the intercept and 0.99 for the slope, with the constraint that *b*_0j_ and *b*_1*j*_ would have a negative correlation of −0.67 (SDs and correlation estimated from Chen et al., 2012 and Goedert et al., 2012). This step produced a negative correlation between slopes and intercepts, as observed in our own data and that of others (Mizuno et al., 2011; Chen et al., 2012). We estimated *e_ij_* of Eq. 2 by randomly drawing from a normal distribution with a mean of 0 and SD of 2.56 (residual variance estimated from Chen et al., 2012 and Goedert et al., 2012).

Thus far, we have described all the components necessary to generate *Y_ij_* based on Eq. 2. However, we added one additional step in the data-generation process. Because slopes observed in our real data had a slight negative skew rather than a normal distribution, we first estimated each subject’s slope as *b*_1_ + *b*_1j_, but then transformed this slope value (Feiveson, 2002) using the method suggested by Fleishman (1978)^1^ to produce slopes that better-resembled the real data. This new, slightly negatively skewed slope was substituted for *b*_1_ + *b*_1*j*_ in Eq. 2. We repeated this data-generation process a minimum of 1000 times for each of the simulation conditions and ran separate MLM and repeated measures ANOVA analyses on each of the generated datasets.

### Analyses and calculations of simulated power and type I error

As with the real data, in the MLM analyses on the generated data, we used maximum likelihood estimation, modeling both random intercepts and random slopes, with an unstructured covariance structure on the random effects. We assumed homogeneity of variance in the residuals. We were as generous as possible to the repeated measures ANOVA, modeling only complete and balanced datasets (equal numbers of subjects in control and treatment groups). For the MLM, we modeled the full-factorial of session and group as the fixed effects, with session as a continuous variable. For the ANOVA, we modeled the full-factorial of session and group, with session as a factor variable, the practice that is common in the field.

For each of the repeated measures ANOVAs and MLMs, we estimated observed *p*-values on the group by session interaction. We estimated *p*-values associated with the uncorrected repeated measures ANOVA (rANOVA) and those using the Greenhouse and Geisser (1959) correction (GG-rANOVA). For the MLM, we estimated *p*-values both using Wald’s *z* and the *F* distribution with between-within *df* (West et al., 2007). As stated earlier, because *z* assumes a large sample, it is assumed that *F* would be a more appropriate distribution to use when testing significance in a relatively small sample. Thus, by estimating and presenting both, we directly tested that assumption here.

It is possible to estimate power and Type I error directly from the proportion of *p*-values below 0.05 on the 1000 datasets generated for each of the simulations described above (e.g., Gueorguieva and Krystal, 2004; Rotello et al., 2008). However, such estimates still demonstrate variability (i.e., the estimates may vary slightly in a different set of 1000 datasets). Therefore, we estimated power and Type I error rates in a second step in which we bootstrapped estimates of power and Type I error: we randomly sampled with replacement samples of size 800 from the 1000 *p*-values and calculated the proportion of *p*-values below 0.05. For effect sizes greater than zero, this proportion is an estimate of power. For effect sizes of zero, this proportion is an estimate of Type I error. We repeated this random sampling process 100 times and present the mean of these 100 bootstrapped samples as the estimates of power and Type 1 error.

^1^Fleishman introduced the following transformation to produce simulated data with a non-normal distribution:

$$Y=a+bX+cX^{2}+dX^{3},$$

where *a*, *b*, *c*, and *d*, are the mean, standard deviation, skewness, and kurtosis, respectively. For our purposes here, we set the mean and standard deviation equal to the mean and standard deviation of the slopes generated via the random normal procedure, but then set *c* = −0.90 and *d* = −0.04, to produce a slight skew in the slopes that mirrored the slight negative skew observed in the individual regression slopes of our real datasets (Chen et al., 2012; Goedert et al., 2012).
